# Rapid Construction of an Infectious Clone of the Zika Virus, Strain ZKC2

**DOI:** 10.3389/fmicb.2021.752578

**Published:** 2021-10-21

**Authors:** Zhiran Qin, Yangyang Chen, Jianhai Yu, Xiaoen He, Xuling Liu, Li Zhu, Qinghua Wu, Chengsong Wan, Bao Zhang, Wei Zhao

**Affiliations:** Guangdong Provincial Key Laboratory of Tropical Disease Research, School of Public Health, Southern Medical University, Guangzhou, China

**Keywords:** Zika virus, homologous recombination, infectious clone, recovery virus, mutation

## Abstract

Zika virus (ZIKV) has had detrimental effects on global public health in recent years. This is because the management of the disease has been limited, in part because its pathogenic mechanisms are not yet completely understood. Infectious clones are an important tool that utilize reverse genetics; these can be used to modify the ZIKV genomic RNA at the DNA level. A homologous recombination clone was used to construct pWSK29, a low copy plasmid that contained sequences for a T7 promoter, the whole genome of ZIKV ZKC2 strain, and a hepatitis delta virus ribozyme. High fidelity PCR was then used to amplify the T7 transcription template. The transcript was then transfected into susceptible cells *via* lipofection to recover the ZIKV ZKC2 strain. Finally, the virulence of rZKC2 was evaluated both *in vitro* and *in vivo*. The rZKC2 was successfully obtained and it showed the same virulence as its parent, the ZIKV ZKC2 strain (pZKC2), both *in vitro* and *in vivo*. The 3730 (NS2A-D62G) mutation site was identified as being important, since it had significant impacts on rZKC2 recovery. The 4015 (NS2A, A157V) mutation may reduce virus production by increasing the interferon type I response. In this study, one of the earliest strains of ZIKV that was imported into China was used for infectious clone construction and one possible site for antiviral medication development was discovered. The use of homologous recombination clones, of PCR products as templates for T7 transcription, and of lipofection for large RNA transfection could increase the efficiency of infectious clone construction. Our infectious clone provides an effective tool which can be used to explore the life cycle and medical treatment of ZIKV.

## Introduction

Zika virus (ZIKV) is an important new mosquito-borne virus that is a member of the *Flavivirus* genus within the Flaviviridae family. ZIKV has an 11-kb positive-sense single-stranded RNA genome which encodes a single polyprotein that can produce ten mature proteins *via* virus and host cell protease processing, including three structural (capsid, C; pre-membrane, prM; and envelope, E) and seven non-structural (NS1, NS2A, NS2B, NS3, NS4A, NS4B, and NS5) proteins (Mukhopadhyay et al., 2005). ZIKV infection is closely related to severe neurological diseases such as Guillain-Barré syndrome in adults and microcephaly in neonates (Cao-Lormeau et al., 2016). Since the large-scale ZIKV epidemic in 2014–2016, it has become a heavy burden on global public health (Heymann et al., 2016). Unfortunately, vaccines and antiviral medications have not yet been developed for ZIKV and its pathogenic mechanisms are not yet fully understood.

Reverse genetic systems can recover viruses containing specific mutations. They are important when studying RNA viruses, as they can modify them at the DNA level, providing a powerful tool for studying the pathogen’s life cycle, and pathogenic and virus–host cell interaction mechanisms. Therefore, constructing infectious clones of ZIKV is an indispensable experimental technique that can be used to help improve our understanding of pathogen biology and investigate related treatment methods.

To date, three main strategies have been utilized for the recovery of viruses (i) The first entails the generation of RNA transcripts from a cDNA clone containing a full-length virus genome, with a prokaryotic RNA polymerase promoter at the 3' end, such as a T7 or SP6 promoter (Shan et al., 2017; Weger-Lucarelli et al., 2017). This involves an *in vitro* transcription and transfection of the transcripts into susceptible cells to recover the virus. Some researchers have also suggested that the hepatitis delta virus ribozyme (HDVr) sequence should be added after the last nucleotide of the ZIKV genome to produce synthetic RNAs with an accurate 3' end (Xu et al., 2021) (ii) The second strategy involves the use of plasmids containing the whole virus genome with a eukaryotic polymerase II-driven promoter at the 5' end; the cytomegalovirus (CMV) promoter has predominantly been used. Furthermore, there should be an HDVr sequence followed by a polymerase II terminator and a polyadenylation signal (pA) at the 3' end. The plasmids are directly transfected into susceptible cells to produce the recovery virus (Schwarz et al., 2016; Lu et al., 2019) (iii) The third method uses infectious subgenomic amplicons (ISA) that separate the virus genome into 3–4 fragments with overlapping sequences and clone them into plasmids, respectively. After co-transfection and *in vivo* homologous recombination, full-length cDNA clones are generated inside the susceptible cells (Atieh et al., 2017; Touret et al., 2018).

Recently, new methods have been used to construct infectious clones. These include the Gibson Assembly method (Pan et al., 2018), which can accelerate the rate of plasmid construction. It is a homologous recombination method that can assemble multiple overlapping DNA fragments easily in a single reaction (Gibson et al., 2009). Furthermore, some chemical transfection methods have also been developed for long length DNA/RNA transfection (Gonzalez et al., 2007). Here, we used the ZIKV ZKC2 strain, one of the earliest strains imported into China, to conduct infectious clone construction. We identified a possible site D62G in the NS2A protein that could be a target for the development of antiviral medicine and another site A157V in the NS2A may be useful to induce a strong interferon type I response. By using homologous recombination, PCR products as the transcription templates, and lipotransfection for large RNA transfection, we achieved relatively faster infectious clone construction.

## Materials and Methods

### Zika Virus and Cells

Zika virus ZKC2 strain (GenBank accession number: KX253996) was generously provided by the pathogenic microorganism testing institute from Guangdong Provincial Center for Disease Control and Prevention. Vero-E6 cells and human umbilical endothelial cells (HUVECs) were maintained in high-glucose Dulbecco’s modified Eagle’s medium (DMEM; Biological Industries, Israel), human brain microvascular endothelial cells (HBMECs) were maintained in RPMI-1640 medium (Biological Industries, Israel), supplemented with 10% fetal bovine serum (FBS; Invitrogen, Carlsbad, United States) and 1% penicillin–streptomycin-amphotericin B solution (Biological Industries) at 37°C with 5% CO_2_. The competent cell XL10 was purchased from Vazyme Biotech Co., Ltd. (Nanjing, China).

### RNA Extraction, Reverse Transcription, and Plasmid Linearization

The genomic RNA of the virus was extracted using the QIAamp^®^ Viral RNA Mini Kit (Qiagen, United States) and then the PrimeScript^™^ II 1st Strand cDNA Synthesis Kit (Takara Biomedical Technology Co., Ltd., Japan) was used to conduct reverse transcription to acquire the cDNA.

The restriction enzymes KpnI (2μl) and SacI (2μl; Takara, Japan) were added to a 50-μl restriction system together with 10 × QuickCut Buffer (5μl), pWSK29 (1μl, 50ng/μl), and ddH_2_O (40μl). After incubation at 37°C for 4h, a gel extraction kit (Omega Bio-tek, Inc., United States) was used to purify the restriction product. All kits were used according to the manufacturer’s instructions.

### Primer Design and Virus Genome Amplification

An online software supported by Takara^1^ was used to design the primers for homologous recombination (Table 1).

**Table 1:** Primers for homologous recombination.

Fragment	Primer	Sequence	Position
1	IN-1F	5' CTCACTATAGGGCGAATTGGACGCGTTAATACGACTCACTATAGG 3'	1–2,489
	IN-1R	5' GATAAGAAGATCAACACTCCCCCTAAGGCC 3'	
2	IN-2F	5' GGAGTGTTGATCTTCTTATCCACAGCCGTCTC 3'	2,490–5,912
	IN-2R	5' CTCCTGGAATCTATGACACGGTCAGCTTTAAAG 3'	
3	IN-3F	5' CGTGTCATAGATTCCAGGAGATGCCTAAAGCCG 3'	5,913–8,638
	IN-3R	5' ACGCTGACCCTTGTGTGGGGGCCTCATAGC 3'	
4	IN-4F	5' CCCCACACAAGGGTCAGCGTCCTCTCTAATAAACG3'	8,639–10,906
	IN-4R	5' CTAAAGGGAACAAAAGCTGGCTCGAGCTTCTCCCTTAGCC 3'	

The Q5 Hot Start High-Fidelity 2X Master Mix (New England Biolabs, lnc. United States) was used to amplify the four fragments containing the homologous sequences. The primer for fragment 1 amplification IN-1F contains a T7 promoter sequence. Fragment 4 was ligated to HDVr using overlapping PCR prior to cloning (forward primer for fragment 4: 5'-ATGCTGTGTCCCGAGGAAG-3'; reverse primer for fragment 4: 5'-GGAGGTGGAGATGCCATGCCGACCCAGACCCATGGATTTCCCC-3'; forward primer for HDVr: 5'-CCGGTGTGGGGAAATCCATGGGTCTGGGTCGGCATGGCATCTC-3'; reverse primer for HDVr: 5'-CTCGAGCTTCTCCCTTAGCCTACCGA-3').

### Homologous Recombination Cloning

**Figure 1:**
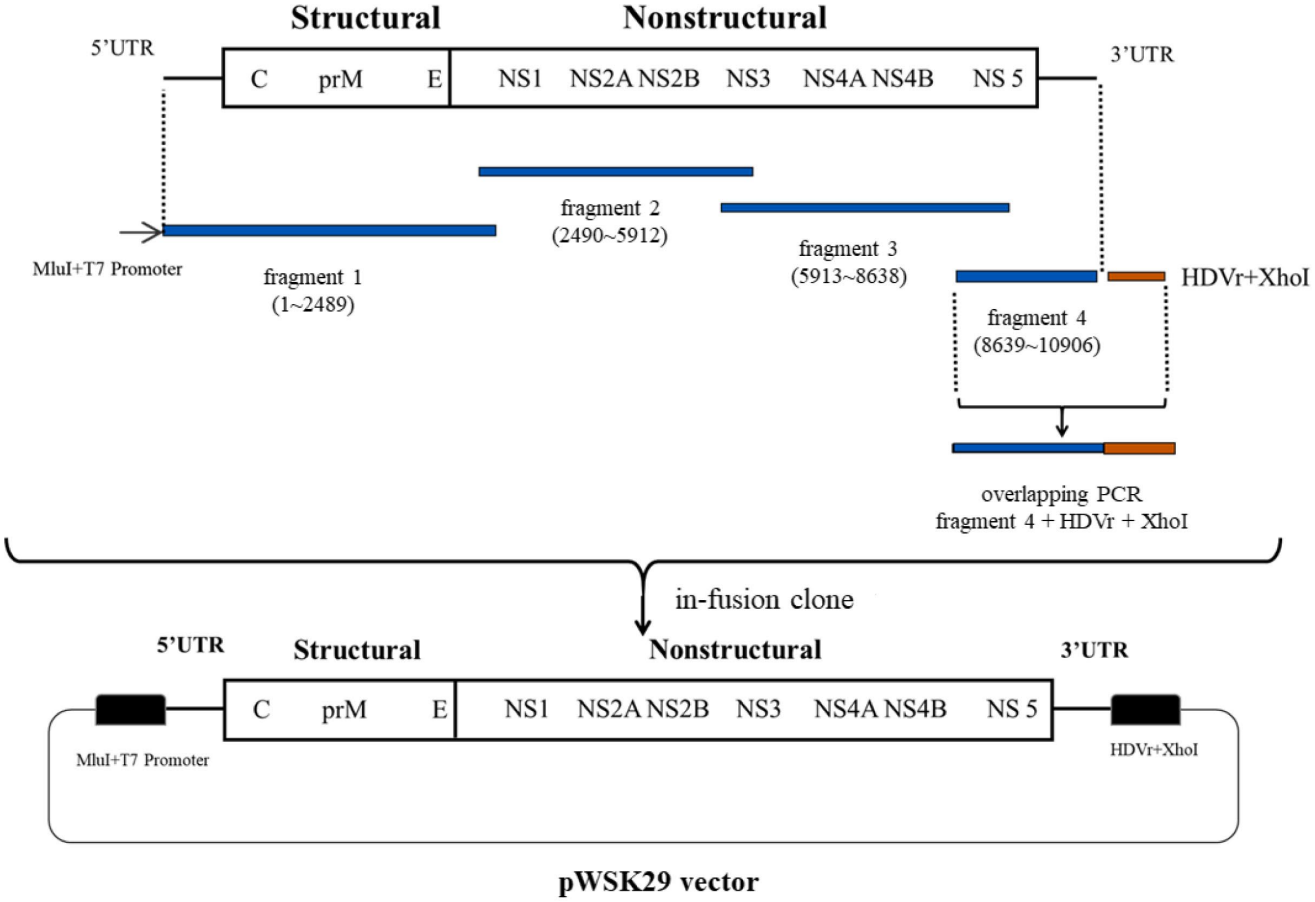
Infectious clone construction procedure for ZIKV ZKC2 strain. Four fragments of PCR product containing a T7 promoter, the whole ZIKV genome, and HDVr were used to clone into linearized plasmid pWSK29 using in-fusion ligation.

The assembly procedure for the ZIKV infectious clone is depicted in Figure 1. The linearized plasmid pWSK29 was used to clone fragments 1 (with T7 promoter), 2, 3, and 4 (with HDVr). A 5X In-Fusion HD Enzyme Premix (4μl), linearized pWSK29 vector (6μl), ddH_2_O (6μl), and the four fragments (1μl, 100ng/μl) were added, respectively and then the reaction system was incubated at 50°C for 15min to perform in-fusion ligation. Finally, 5μl of the ligated plasmid was transformed into the competent cell line XL10. The constructed pWSK29 vector containing the target sequences was extracted using a TaKaRa MiniBEST Plasmid Purification Kit Ver.4.0 (Takara) for T7 transcription template amplification using the High-Fidelity PCR Master Mix (New England Biolabs) with a full-length primer (from T7 promoter to HDVr; forward primer: 5'-TAATACGACTCACTATAGGGAGTTGTTGATCTGTGTG-3'; reverse primer: 5'-CTTCTCCCTTAGCCTACCGAAGTAGCCCAGGT-3').

### T7 Transcription and Virus Genomic RNA Transfection

After PCR product purification using a E.Z.N.A Gel Extraction Kit (Omega), the template DNA was used to perform T7 transcription using a mMESSAGE mMACHINE^®^ High Yield Capped RNA T7 Transcription Kit (Life Technologies, United States). Then the synthetic viral genome RNA was purified using a MEGAclear^™^ Purification for Large Scale Transcription Reaction Kit (Life Technologies) and transfected into Vero cells using a TransIT^®^-mRNA Transfection Kit (Mirus, United States). All kits were used according to the manufacturer’s instructions.

### Virus Quantification

For the virus plaque assay, virus supernatants were diluted serially 10-fold in DMEM, and a 100μl aliquot of each dilution was added to Vero cell monolayers in 24-well plates. After 2-h incubation with the cells at 37°C and 5% CO_2_, a 3-ml DMEM overlay containing a final concentration of 1.2% methylcellulose and 2% FBS was added to all wells. Plaques were counted 6days after the addition of the overlay. Infectious virus titer is reported as plaque forming units (PFU)/ml. For the qPCR, the ZIKV-specific primers (probe: 5'-FAM-TGGCAGCTYCTTTATTTCCACARAAG-BHQ-3'; ZIKV-F 5'-GGCRTTRGCCATCAGTCG-3'; ZIKV-R 5'-ATGGAGCATCCGKGAGACT-3') designed by our group were used for qRT-PCR. Detection of the amplification was carried out using the Evo M-MLV RT Master Mix and Pro Taq HS Probe Premix (ACCURATE BIOLOGY, Hunan, China) following the manufacturer’s protocol. PCR amplification was conducted in 96-well plates using the Roche LightCycler 96 System. A standard curve was used for absolute quantification using a standard plasmid containing the pMD^®^-18T vector (Takara), which comprised the target gene.

### Indirect Immunofluorescence Assays

Cells were washed with PBS twice and fixed in 100% ethanol at 4°C for 20min. After washing with the wash buffer containing 0.2% Triton X-100 in PBS for 10min, the cells were treated using a blocking buffer containing 3% BSA in PBS at 37°C for 1h. Then the cells were treated with a mouse monoclonal antibody 4G2 overnight and washed three times with PBS (5min for each wash). The cells were then incubated in the dark with FITC-conjugated goat anti-mouse IgG (Proteintech, United States) for 1h, after which the cells were washed three times with PBS. In the end, 40μl of Hoechst stain (1×2000) was added for nuclear staining. Fluorescence images were observed under a fluorescence microscope equipped with a video documentation system (Nikon, Japan).

### Animal Test and Histopathological Analysis

The animal study design was approved by the Laboratory Animal Ethics Committee of Southern Medical University (approval number: 2012-041) and animal care was in accordance with the institutional guidelines. Animal model in this study have been established by our team in a previous study (Yu et al., 2017). One-day-old suckling mice were challenged with 5×10^6^ PFU ZIKV using a subcutaneous multipoint injection route. The suckling mice were divided into three group, including rZKC2 group, pZKC2 group and control group (*N*=10 for C67BL/6 suckling mice, *N*=12 for Kunming suckling mice). The brains of the suckling mice were then harvested, and prior to staining for histopathology, they were fixed in 10% buffered formalin and processed for histopathology by Servicebio Biotechnology Co., Ltd. (Wuhan, China).

### Site-Directed Mutagenesis Analysis

The three-dimensional structure of residue 55–74 and 151–170 of NS2A protein was predicted by the online protein prediction platform C-I-TASSER^2^ with submitting number CIT2120 (pZKC2)^3^ and CIT2141 (rZKC2)^4^. The NS1 protein residues 211–230 before and after mutation were predicted using the PC software Discovery Studio 2.5 (DS2.5) by Homology Modeling. A Q5^®^ Site-Directed Mutagenesis Kit (NEB) was used according to the manufacturer’s instructions to introduce mutations with the primers, 3,730-F 5'-ATTTTGATGGgTGCCACCTTC-3' and 3730-R 5'-TGCAAGCTTAGCCAGGTC-3', designed using the NEB online software.^5^

### *In vitro* Immune Response Detection

HBMECs and HUVECs (5×10^4^ per well) were seeded onto 24-well plates and infected with rZKC2 and pZKC2 (MOI=1). After 1h incubation at 37°C, the virus inoculum was removed and replaced with a growth medium containing 2% FBS. Cell lysates were collected at 6, 24, and 48h post-infection for viral RNA quantification using the SteadyPure Universal RNA Extraction Kit II (Accurate Biology), Evo M-MLV Mix Kit with gDNA Clean for qPCR (Accurate Biology), and SYBR^®^ Green Premix Pro Taq HS qPCR Kit (Accurate Biology). Except for the primer for *GAPDH* as the reference gene (5'-GTGGACCTGACCTGCCGTCT-3', 5'-GGAGGAGTGGGTGTCGCTGT-3'), the other genes (*DDX58*, *IFIT1*, *ISG15*, *IFNB1*, *TNF*-α, and *IL-6*) were identified from the PrimerBank database,^6^ a public resource for qPCR primers.

### Statistical Analysis

All data were analyzed with GraphPad Prism v6 software (San Diego, CA, United States). Data are expressed as the mean±SD. Two independent sample *t*-tests and one-way ANOVA were used for statistical analysis using SPSS 20.0 (Chicago, IL, United States), and a *p*<0.05 indicated statistical significance. Comparison of Kaplan–Meier survival curves between the animal experiment groups was performed using log-rank analysis.

## Results

### Genome of Zika Virus Strain ZKC2 Was Successfully Ligated Into the pWSK29 Vector

The entire genome of the ZIKV ZKC2 strain was divided into four PCR fragments containing homologous sequences for homologous recombination. The T7 promoter was introduced into the upstream primer of fragment 1. Fragment 4 was ligated to Hepatitis D ribozyme (HDVr) using overlapping PCR. Subsequently, the T7 promoter, the full-length genome of the ZIKV ZKC2 strain, and HDVr, were ligated into the PWSK29 low copy vector using in-fusion homologous recombination (Figure 1). Finally, the constructed plasmids were transformed into XL10 competent cells.

Compared with the pZKC2 strain, the rZKC2 sequence had 6 mutation sites: G3149A (NS1, M220I), D3730G (NS2A, G62D), C4015T (NS2A, A157V), C5633T (NS3, synonymous mutation), 5744 (NS3, synonymous mutation), and 7007 (NS4B, synonymous mutation; Supplementary Material, the whole sequence of ZIKV ZKC2 strain infectious clone)

**Figure 2:**
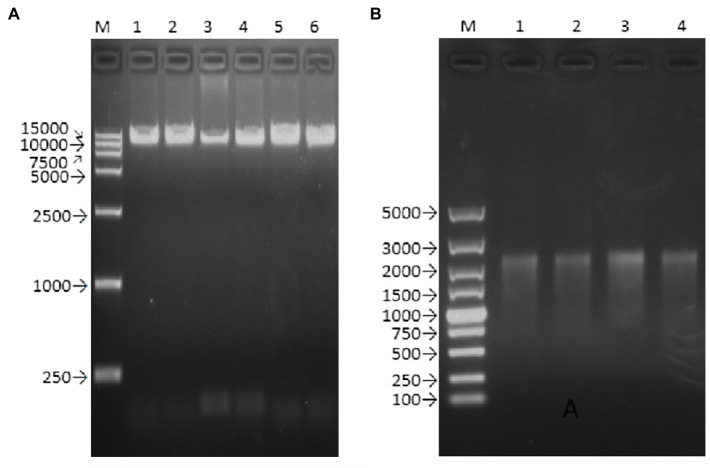
Agarose gel electrophoresis of PCR product and transcript of rZKC2. **(A)** Full-length PCR product containing the whole ZIKV genome (with a T7 promoter and HDVr at the end of the 5' UTR and 3' UTR, respectively; **(B)** Full-length transcript from the purified full-length PCR product.

The Q5 superfidelity enzyme was used to amplify the DNA templates (including the T7 promoter, the entire genome of the Zika virus ZKC2 strain, and HDVr) for *in vitro* transcription (Figure 2A). The RNA was then transfected into Vero cells *via* liposome transfection. The length of the transcript RNA was confirmed using RNA electrophoresis (Figure 2B). We used a DL5000 DNA marker, and the length of the target RNA was expected to be 2,000–3,000bp, according to a previous study (Lu et al., 2019).

### Mutation D62G Has Significant Impact on Virus Recovery

**Figure 3:**
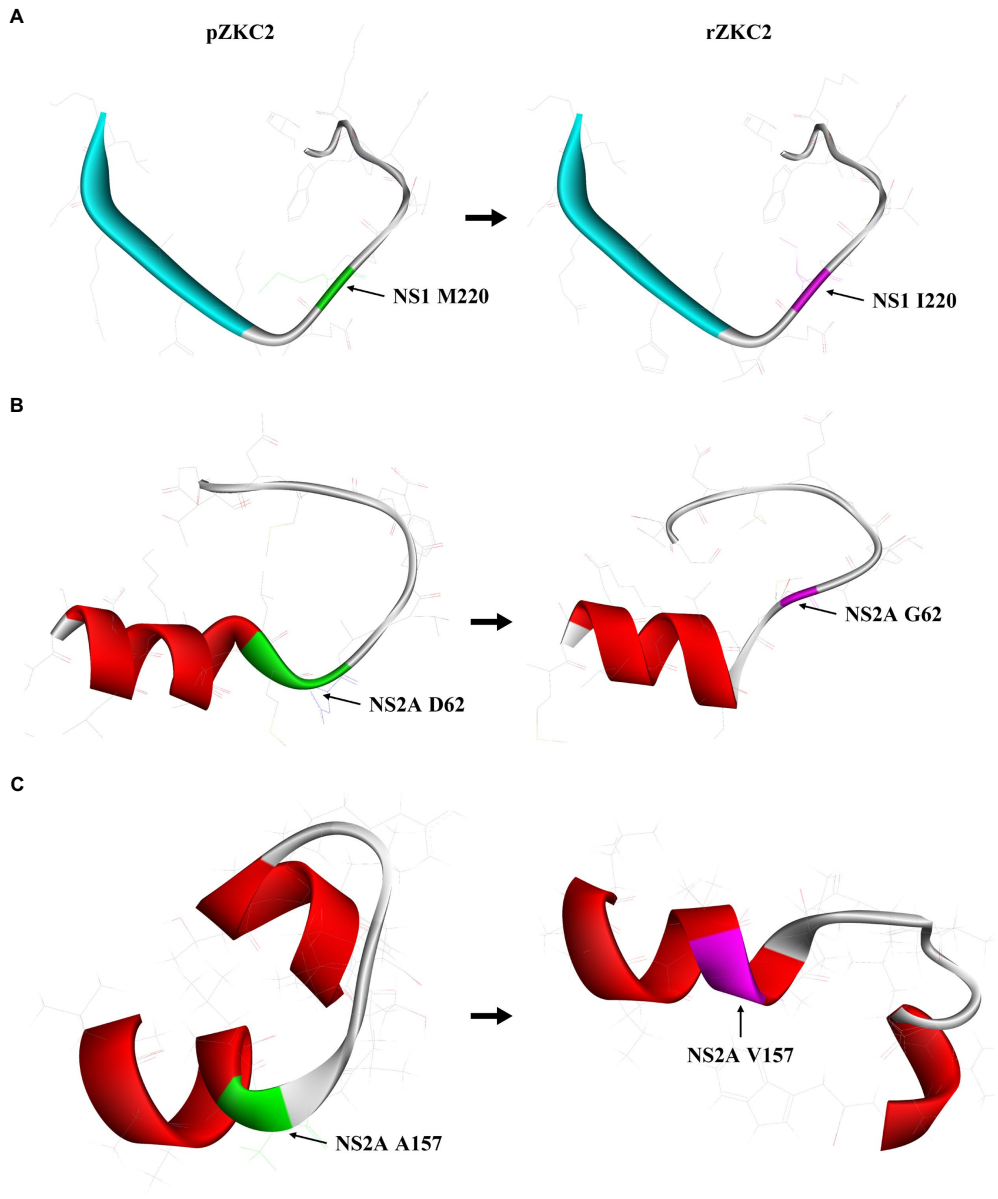
The impact of three mutation on its protein structure. **(A)** 3D structure analysis of mutation M220I of NS1 protein residue 211–230. **(B)** 3D structure analysis of mutation D62G of NS2A protein residue 55–74. **(C)** 3D structure analysis of mutation A157V of NS2A protein residue 151–170.

At first no recovery virus was obtained and the three alterations resulting in non-synonymous mutations were analyzed selectively. The mutation G3149A (NS1, M220I) had no significant impact on the structure of NS1 (Figure 3A). The NS2A protein 55–74 amino acid extra-membrane protein simulation results showed that the mutation D3730G (NS2A, G62D) may lead to shorter trans-membrane domain, making the structure less stable (Figure 3B), and suggesting that this mutation may have a significant impact on virus recovery. In contrast, C4015T (NS2A, A157V) mutation resulted in trans-membrane domain extension in its structure, making NS2A protein more stable (Figure 3C).

**Figure 4:**
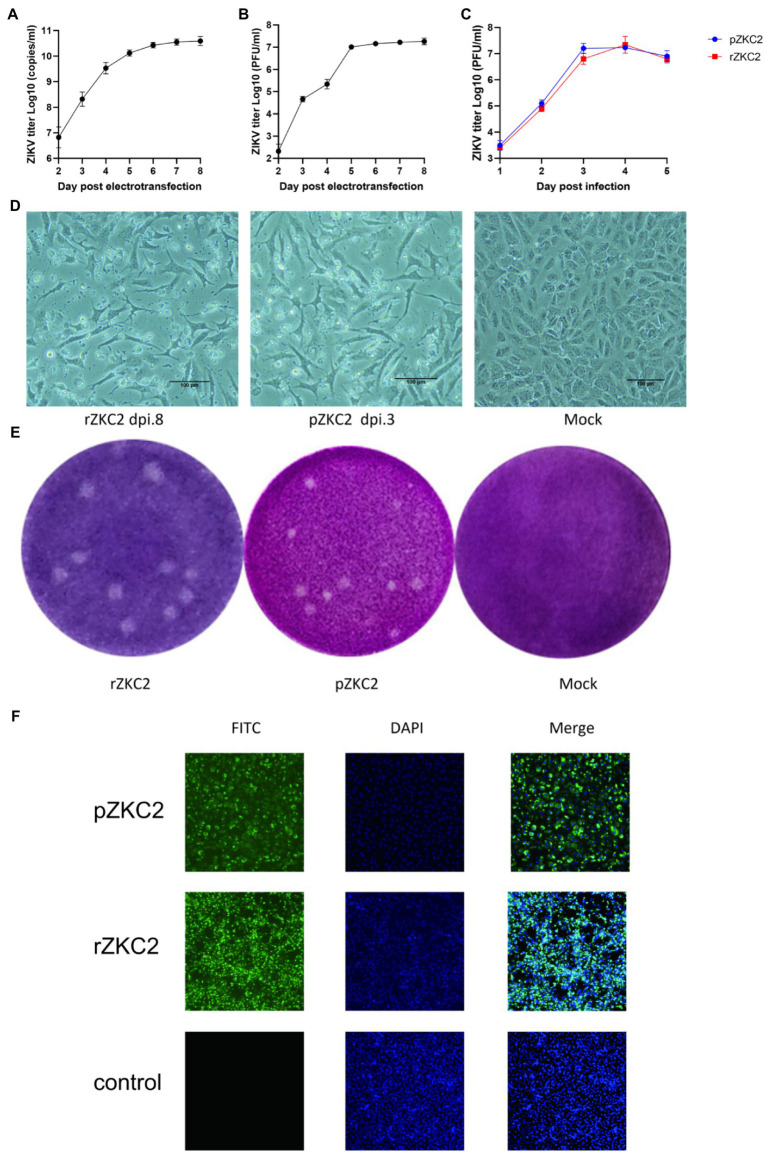
The obtained rZKC2. **(A)** The viral copy number in the supernatant after eletrotransfection. **(B)** The infectious virus count in the supernatant after eletrotransfection. **(C)** Growth kinetics curves of the rZKC2 and pZKC2. **(D)** Cytopathic effect of the rZKC2 and pZKC2. (E) Plaque assay results of the rZKC2 and pZKC2. (F) IFA results of the rZKC2 and pZKC2.

After the 3,730 site-directed back mutagenesis and T7 transcription, over 50% of the RNA-transfected cells showed significant cytopathic effects (CPE) at 8days after infection (dpi 8; Figures 4A,B,D). No further mutations were generated in the plasmid and the recovery virus for five consecutive generations (data not shown). Both rZKC2 and pZKC2 showed the same plaque size and were verified by IFA (Figures 4E,F).

On the third day after electroporation, IFA was used to detect the expression of the ZIKV envelope protein. The results showed a large amount of green fluorescence upon the analysis of the pZKC2- and rZKC2-infected cells, indicating that rZKC2 was successfully rescued, and that the E protein could be replicated and expressed in the Vero cells (Figure 4F).

### Recovery Virus Shows the Same Virulence as the Parental Virus in the Immunodeficient Host

Virus titer in the supernatant was detected 1–5 dpi. Growth kinetic curves indicated that the virulence of the recovery virus was similar to that of the parental virus *in vitro*. The peak titer of the recovery virus in the supernatant of the Vero cells was 10^10^ copies/ml and 10^7^ PFU/ml (Figure 4C).

**Figure 5:**
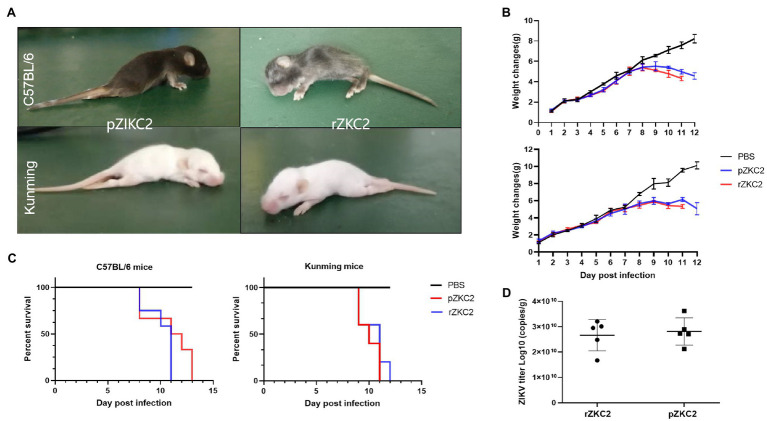
*In vivo* tests for the rZKC2 and pZKC2 in the neonatal mice. **(A)** Symptoms of suckling mice after infection; **(B)** Weight change; **(C)** Survival curves; and **(D)** Virus titer in the brains of the C57BL/6 and Kunming suckling mice infected by pZKC2 and rZKC2. The number (n) of mice used was 10 for C57BL/6 neonatal mice and *n*=12 for Kunming neonatal mice. Log-rank test (*p*>0.05) was used to compare the survival curve data.

We used C57BL/6 and a Kunming suckling mouse model constructed by our group in a previous study (Yu et al., 2017) to confirm the virulence of rZKC2. After administering rZKC2 to C57BL/6 and the Kunming one-day-old neonatal mice using multi-point subcutaneous injections, all the neonatal mice showed lower limb weakness, slow movements, hump, tremor, abnormal gait, and weight loss 5 dpi (Figure 5A), and all mice died 9–12 dpi, with a mortality rate of 100% (Figure 5C).

The body weights of the infected C57BL/6 suckling mice decreased significantly at 9–13 dpi (*p*<0.05), when compared with the control group. The Kunming suckling mice showed the same changes at 9–11 dpi (*p*<0.05; Figure 5B). We also detected the virus titer in the mouse brain and data showed that both pZKC2 and rZKC2 in the brain reached an order of magnitude of 10^10^ copies/g (*p*>0.05; Figure 5D).

**Figure 6:**
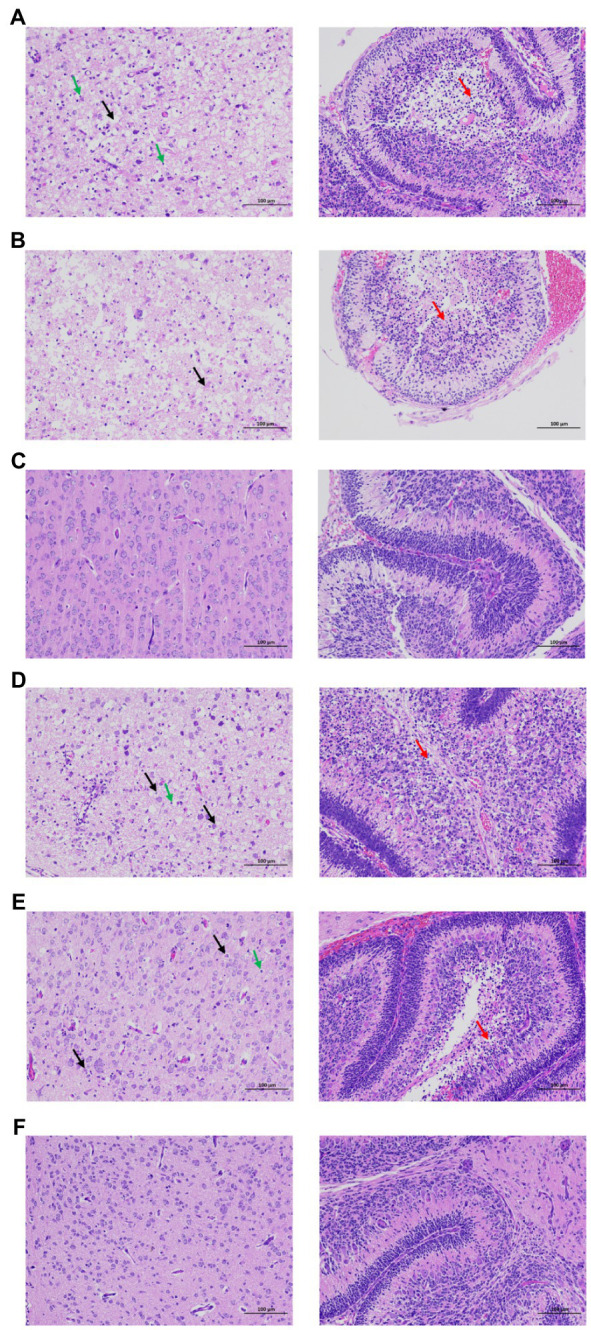
Brain pathological analysis of the C57BL/6 and Kunming neonatal mice infected with rZKC2 and pZKC2. Both moribund C57BL/6 and Kunming suckling mice had massive neuronal necrosis in the brain (black arrows), with pyknotic, fragmented, and obliterated nuclei, leaving empty spaces in the cytoplasm. Furthermore, minor neutrophil and lymphocyte infiltration (green arrows) was observed. In the cerebellum, more granular layers of cells were focally necrotic, and the nuclei were pyknotic and fragmented, and disappeared subsequently (red arrows). The morphology of nerve cells in the control group was normal, and no inflammatory cell infiltration was observed. **(A)** C57BL/6 neonatal mice infected by rZKC2; **(B)** C57BL/6 neonatal mice infected by pZKC2; **(C)** C57BL/6 neonatal mice control group. **(D)** Kunming neonatal mice infected by rZKC2; **(E)** Kunming neonatal mice infected by pZKC2. **(F)** Kunming neonatal mice control group. Results from one independent experiment using six pZKC2 and rZKC2 infected neonatal mice brains and three uninfected controls were used.

After infection of rZKC2 and pZKC2, both moribund C57BL/6 and Kunming suckling mice had massive neuronal necrosis in the brain, with pyknotic, fragmented, and obliterated nuclei, leaving empty spaces in the cytoplasm. Furthermore, minor neutrophil and lymphocyte infiltration was observed. In the cerebellum, more granular layers of cells were focally necrotic, and the nuclei were pyknotic and fragmented, and disappeared subsequently (Figure 6).

Both the rZKC2- and pZKC2-infected suckling mice produced the same disease process with similar mortality rates, and the same virus titers with the same orders of magnitude in the brain, suggesting that rZKC2 virulence was similar to that of the pZKC2 *in vivo*.

### Recovery Virus Resulted in a Stronger Interferon Type I Response in HBMECs and HUVECs

**Figure 7:**
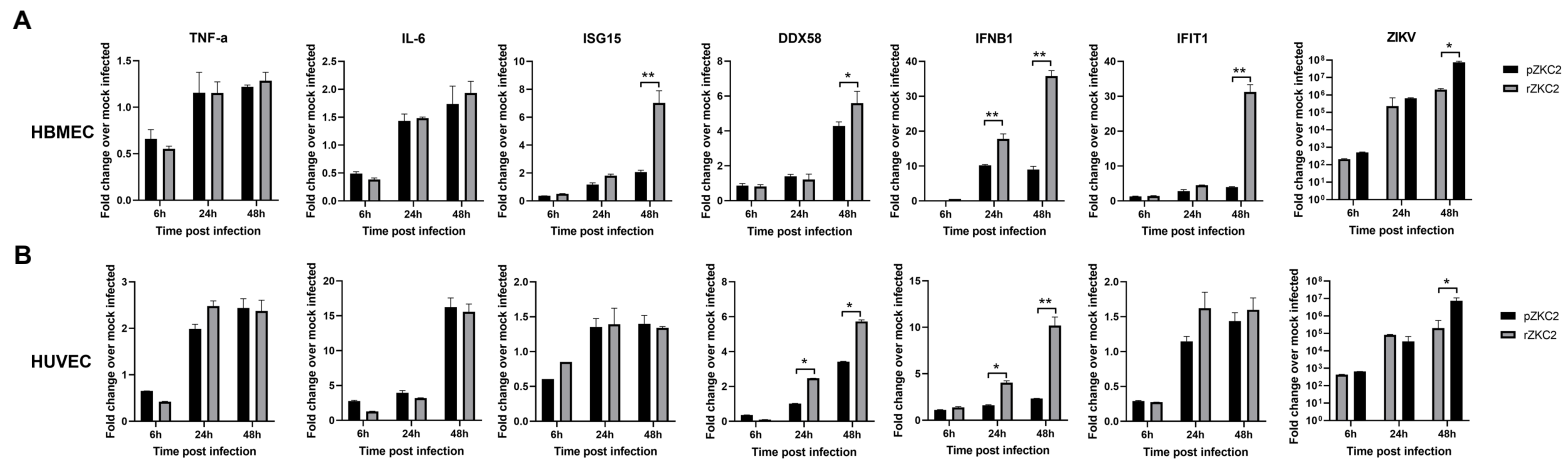
The immune response of rZKC2 and pZKC2 in HBMEC and HUVEC. **(A)** Fold change in transcript levels of the TNF-α, IL-6, ISG15, DDX58, IFNB1, IFIT1 and ZIKV viral RNA (NS5 gene) in HBMEC cells at different time points post-infection relative to mock-infected control. **(B)** Fold change in transcript levels of the same genes in HUVEC cells at different time points post-infection relative to mock-infected control. The values are representative of three repeat experiments. ^*^p<0.05, ^**^p<0.01.

Vero-E6 are immunocompromised cells, and therefore, immunocompetent cells including HBMECs and HUVECs were used to test the immune response to rZKC2 and pZKC2. The transcript levels of the interferon-stimulated genes (ISGs) *DDX58*, *IFIT1*, *ISG15*, and *IFNB1* were evaluated to determine the interferon type I response. Greater transcript levels for all four ISGs were observed in HBMECs, and the greater expression of *DDX58* and *IFNB1* was detected in HUVECs. Similar transcript levels for the inflammatory cytokines TNF-α and IL-6 were observed in both HBMECs and HUVECs. Because of the greater ISGs signal, lower viral replication efficiencies were observed 48h post-infection in HBMECs and HUVECs (Figure 7).

## Discussion

There have been several large-scale ZIKV outbreaks around the world. Like other *flaviviruses*, ZIKV poses a great threat to human health and has created a large-scale global public health challenge. Unfortunately, at present there is no specific treatment or vaccine available for ZIKV infection and its pathogenic mechanisms are unclear (Ferraris et al., 2019). It is thus important that we continue to research and improve our understanding of the ZIKV genome. The ZIKV genome is a single-stranded RNA virus and its genomic RNA functions as messenger RNA and can cause infection (Rodriguez et al., 2019). One strategy to solve this problem is to use reverse genetic systems to conduct mutation studies and test medications relatively easily, by editing the genome of the RNA virus at the DNA level (Ávila-Pérez et al., 2018). In our study, one of the earliest ZIKV strains, which was imported into China in 2016, was used to construct an infectious clone. It is one of the standard strains used to perform sequence analyses of the ZIKV.

To date, three methods to construct infectious clones have been utilized and were described in the Introduction. Each method has its advantages and disadvantages. For strategy (i), the viral RNA is directly transfected into cells, and usually has a high recovery rate due to the production efficiency of the virus (Aubry et al., 2015). However, it requires additional *in vitro* RNA transcription steps, and genome instability of the cDNA clones in bacteria has been reported (Widman et al., 2017). To address these problems, we used an RNase cleaner to inactivate the RNase on the surface of the pipettor and the other furniture prior to conducting the RNA experiments. Furthermore, RNase-free consumables were also used. After T7 transcription, the RNase inhibitor was used in the transcript. For strategy (ii), the plasmid containing the viral genome is directly transfected into the cells so that *in vitro* transcription is not required (Liu et al., 2017). However, the problem of genome instability remains. In strategy (iii) plasmids are rapidly and easily constructed but nonhomogeneous populations can be produced due to errors in the recombination events (Tsetsarkin et al., 2016). In our study, we used strategy (i) and inserted three adenines in between the T7 promoter and the 5' end of the ZIKV to achieve high efficiency levels during *in vitro* transcription. The results showed that rZKC2 was successfully rescued and suggests that the three inserted adenines did not affect the secondary structure of the rZKC2 genomic RNA. At the same time, a HDVr sequence was ligated to the 3' end of the ZIKV genome to obtain an accurate 3' end. We also tried to use different methods during the construction of the infectious clones to achieve a faster virus recovery.

The basic methodology of this investigation was based on classical methods, which were then combined with more modern methods. Ultimately, we used three methods to improve the efficiency of infectious clone construction. During the plasmid construction, we used homologous recombination clones instead of a classical restriction enzyme ligation method, as they did not require restriction site cutting and could increase the chance of successful plasmid construction. A one-time in-fusion clone was required in our study with a 20% positive clone rate. We selected 10 clones after the transformation of the XL10 competent cells and used the 4 PCR fragments to carry out primary selection prior to sequencing. The results showed that 2 of the 10 clones were positive for all 4 fragments, which contained the complete ZIKV genome.

After plasmid construction, we used purified PCR products containing a T7 promoter, the whole ZIKV genome, and HDVr to conduct the T7 transcription. This method required a high-fidelity PCR enzyme to minimize the mutation rate during the PCR process. Based on the results of our literature search, the use of a PCR product as a T7 transcription template has not been reported earlier since the length of the amplified product may hinder its accurate amplification. We examined the accuracy of our method over five rounds and the results showed that no additional mutations were generated. In contrast to the methodology of restriction enzyme cutting and purifying, the use of purified PCR products could obtain high concentrations of the transcription template in a shorter time without using a large centrifuge to conduct large scale plasmid extraction. Electro-transfection is the most classical method used to conduct large scale RNA transfection. The use of the chemical transfection method for transfecting viral genomic RNA during the acquisition of the recovery virus has not been reported in the literature. In our study, however, we used a chemical transfection method for long RNA transfection, which was cheaper according to our accounts, but were still able to recover the virus.

Based on the results of this study, we recommend the following procedure for developing infectious clones: (i) Homologous recombination should be performed during plasmid construction (ii) Purified PCR products synthesized using a high fidelity polymerase should be employed as a transcription template to obtain viral genomic RNA (iii) Chemical transfection for long-length RNA should be performed for transfecting RNA into the susceptible cells. Significantly, time-consuming traditional restriction enzyme digestion, further processing using ligation, high-speed centrifugation for large-scale plasmid extraction, and relatively expensive electroporation methods can be avoided using the methodology described in this study.

When compared with strategy (iii), our method showed a relatively higher mutation rate, which may lead to failures when trying to recover the virus. Six mutations were identified in our infectious clone, including three non-synonymous mutations, and two of them were in the NS2A protein gene [3730 (NS2A-D62G) and 4015 (NS2A-V157A)]. The NS2A protein of flavivirus is a 22-kDa hydrophobic protein, which plays an important role in virus replication, assembly, secretion, and immune evasion (Márquez-Jurado et al., 2018). The topological model of the NS2A protein constructed using bioinformatics indicates that the NS2A protein contains a single trans-membrane domain across the endoplasmic reticulum membrane and that six membrane-related regions and the sites that affect RNA replication or virus assembly may be concentrated in the outer membrane region (Zhang et al., 2019a). In the outer membrane region, the ZIKV NS2A protein recruits genomic RNA, the structural protein prM/E complex, and the NS2B/NS3 protease complex to the virion assembly site. After processing the C-prM-E polyprotein, NS2A presents viral RNA to the structural proteins for virion assembly (Zhang et al., 2019b). Mutation 3730 (NS2A-D62G) is in the outer membrane region while 4015 (NS2A-V157A) is in the inner membrane region. Thus we first analyzed mutation 3,730, and found that it may lead to an shorter trans-membrane domain, which makes the structure less stable and may lead to failures while trying to obtain rZKC2. After back mutations of the 3,730 mutation, rZKC2 was successfully obtained. Our results indicate that the 3730 (NS2A-D62G) mutation is important for rZKC2 recovery. It could also possibly be a target site for the development of antiviral medications.

After constructing rZKC2, Vero-E6 cells were used to evaluate the replication efficiency of rZKC2; however, Vero-E6 is an interferon type I deficient cell line (Earley and Johnson, 1988). Therefore, immunocompetent cells including HBMECs and HUVECs were used to analyze the immune response to rZKC2 and pZKC2. HBMECs and HUVECs have been used to analyze whether the virus can cross the blood–brain barrier (Papa et al., 2017) and placental barrier (Peng et al., 2018), respectively. The innate immune response, especially the IFN pathway, is activated due to the presence of viral RNA, which is pathogen-associated molecular pattern (PAMP) that are recognized by pattern recognition receptors (PRRs; Green et al., 2014). However, host immune responses are weakened due to the degradation of STAT1 and STAT2 transcription factors that initiate the transcription of ISGs by NS2A during ZIKV infection (Fanunza et al., 2021). Hence, we examined the ISGs transcript levels for *DDX58*, *IFIT1*, *ISG15*, and *IFNB1*, and analyzed the transcript levels of inflammatory cytokines including *TNF-α* and *IL-6* that are associated with severe symptoms during ZIKV infection (Abdalla et al., 2018). We observed that rZKC2 infection resulted in greater expression of ISGs compared to pZKC2, whereas the level of inflammatory cytokines remained comparable to that for pZKC2 in HBMECs and HUVECs. Because of the greater ISGs signal, lower viral replication efficiencies were observed. Based on the analysis for protein structure, the mutation A157V in NS2A resulted in a different tertiary structure compared to the parental virus, which may impair the function of NS2A. Therefore, we hypothesize that the A157V mutation impairs the function of NS2A, thereby inducing a greater innate immune response and inhibiting rZKC2 replication. However, further studies are required to analyze the impact of the NS2A A157V mutation on ZIKV replication and host immune responses.

## Conclusion

One of the earliest ZIKV strains, ZKC2, was imported into China in 2016 and was used to conduct infectious clone construction. The NS2A D62G mutation has huge impacts for obtaining recovery virus and may become a target for antiviral medications in the future. NS2A A157V mutation may induce a greater interferon type I response. Improved methods for the process of infectious clone construction were developed. The results show that the use of homologous recombination clones during plasmid construction, PCR products as templates for T7 transcription, and lipotransfection for large RNA transfections can be utilized for cheaper and faster infectious clone construction, while using the RNA transcripts from a cDNA clone strategy. Further studies are warranted to evaluate the potential impact of NS2A A157V mutation on ZIKV virulence.

## Data Availability Statement

The original contributions presented in the study are included in the article, further inquiries can be directed to the corresponding authors.

## Ethics Statement

The animal study was reviewed and approved by Laboratory Animal Ethics Committee of Southern Medical University.

## Author Contributions

All authors contributed to the study design and data analysis. ZQ and WZ contributed to the manuscript preparation. XL contributed to the additional study during interactive review.

## Funding

This research was supported by the National Key Research and Development Program of China (2018YFC1602206), the National Natural Science Foundation of China (81730110 and 31470271), Yangjiang Science and Technology Program key projects (2019010), the Basic Research Project of Key Laboratory of Guangzhou (No. 202102100001) and Guangdong Science and Technology Program key projects (2018B020207006).

## Acknowledgments

We would like to express our sincere gratitude to the pathogenic microorganism testing institute from Guangdong Provincial Center for Disease Control and Prevention for providing us ZIKV ZKC2 strain. Also, we would like to thank Editage (www.editage.cn) for English language editing.

## Conflict of Interest

The authors declare that the research was conducted in the absence of any commercial or financial relationships that could be construed as a potential conflict of interest.

## Publisher’s Note

All claims expressed in this article are solely those of the authors and do not necessarily represent those of their affiliated organizations, or those of the publisher, the editors and the reviewers. Any product that may be evaluated in this article, or claim that may be made by its manufacturer, is not guaranteed or endorsed by the publisher.

## Footnotes

^1^https://www.takarabio.com/learning-centers/cloning/primer-design-and-other-tools?citemId=https%3a%2f%2fwww.takara-bio.co.jp%2finfusion_primer%2finfusion_primer_form.php&section=16260&xxheight=1800

^2^https://zhanggroup.org/C-I-TASSER/

^3^https://zhanggroup.org/C-I-TASSER/output/CIT2120/

^4^https://zhanggroup.org/C-I-TASSER/output/CIT2141/

^5^https://NEBaseChanger.neb.com

^6^https://pga.mgh.harvard.edu/primerbank/index.html
