# Supplementary material for: Rapid Construction of an Infectious Clone of the Zika Virus, Strain ZKC2
Source: Front Microbiol. 2021 Oct 21;12:752578. doi: 10.3389/fmicb.2021.752578 (PMC8567171; doi:10.3389/fmicb.2021.752578)
Supplement: Supplementary file 1 [file Data_Sheet_1.docx]

The whole sequence of ZIKV ZKC2 strain infectious clone

Black: pWSK29 vector sequence

Yellow: T7 promoter sequence

Red: ZIKV ZKC2 strain sequence

Blue: HDVr sequence

TCTGACGCTCAGTGGAACGAAAACTCACGTTAAGGGATTTTGGTCATGAGATTATCAAAAAGGATCTTCACCTAGATCCTTTTAAATTAAAAATGAAGTTTTAAATCAATCTAAAGTATATATGAGTAAACTTGGTCTGACAGTTACCAATGCTTAATCAGTGAGGCACCTATCTCAGCGATCTGTCTATTTCGTTCATCCATAGTTGCCTGACTCCCCGTCGTGTAGATAACTACGATACGGGAGGGCTTACCATCTGGCCCCAGTGCTGCAATGATACCGCGAGACCCACGCTCACCGGCTCCAGATTTATCAGCAATAAACCAGCCAGCCGGAAGGGCCGAGCGCAGAAGTGGTCCTGCAACTTTATCCGCCTCCATCCAGTCTATTAATTGTTGCCGGGAAGCTAGAGTAAGTAGTTCGCCAGTTAATAGTTTGCGCAACGTTGTTGCCATTGCTACAGGCATCGTGGTGTCACGCTCGTCGTTTGGTATGGCTTCATTCAGCTCCGGTTCCCAGCGATCAAGGCGAGTTACATGATCCCCCATGTTGTGCAAAAAAGCGGTTAGCTCCTTCGGTCCTCCGATCGTTGTCAGAAGTAAGTTGGCCGCAGTGTTATCACTCATGGTTATGGCAGCACTGCATAATTCTCTTACTGTCATGCCATCCGTAAGATGCTTTTCTGTGACTGGTGAGTACTCAACCAAGTCATTCTGAGAATAGTGTATGCGGCGACCGAGTTGCTCTTGCCCGGCGTCAATACGGGATAATACCGCGCCACATAGCAGAACTTTAAAAGTGCTCATCATTGGAAAACGTTCTTCGGGGCGAAAACTCTCAAGGATCTTACCGCTGTTGAGATCCAGTTCGATGTAACCCACTCGTGCACCCAACTGATCTTCAGCATCTTTTACTTTCACCAGCGTTTCTGGGTGAGCAAAAACAGGAAGGCAAAATGCCGCAAAAAAGGGAATAAGGGCGACACGGAAATGTTGAATACTCATACTCTTCCTTTTTCAATATTATTGAAGCATTTATCAGGGTTATTGTCTCATGAGCGGATACATATTTGAATGTATTTAGAAAAATAAACAAATAGGGGTTCCGCGCACATTTCCCCGAAAAGTGCCACCTAAATTGTAAGCGTTAATATTTTGTTAAAATTCGCGTTAAATTTTTGTTAAATCAGCTCATTTTTTAACCAATAGGCCGAAATCGGCAAAATCCCTTATAAATCAAAAGAATAGACCGAGATAGGGTTGAGTGTTGTTCCAGTTTGGAACAAGAGTCCACTATTAAAGAACGTGGACTCCAACGTCAAAGGGCGAAAAACCGTCTATCAGGGCGATGGCCCACTACGTGAACCATCACCCTAATCAAGTTTTTTGGGGTCGAGGTGCCGTAAAGCACTAAATCGGAACCCTAAAGGGAGCCCCCGATTTAGAGCTTGACGGGGAAAGCCGGCGAACGTGGCGAGAAAGGAAGGGAAGAAAGCGAAAGGAGCGGGCGCTAGGGCGCTGGCAAGTGTAGCGGTCACGCTGCGCGTAACCACCACACCCGCCGCGCTTAATGCGCCGCTACAGGGCGCGTCCCATTCGCCATTCAGGCTGCGCAACTGTTGGGAAGGGCGATCGGTGCGGGCCTCTTCGCTATTACGCCAGCTGGCGAAAGGGGGATGTGCTGCAAGGCGATTAAGTTGGGTAACGCCAGGGTTTTCCCAGTCACGACGTTGTAAAACGACGGCCAGTGAGCGCGCGTAATACGACTCACTATAGGGCGAATTGGACGCGTTAATACGACTCACTATAGGGAGTTGTTGATCTGTGTGAATCAGACTGCGACAGTTCGAGTTTGAAGCGAAAGCTAGCAACAGTATCAACAGGTTTTATTTTGGATTTGGAAACGAGAGTTTCTGGTCATGAAAAACCCAAAAAAGAAATCCGGAGGATTCCGGATTGTCAATATGCTAAAACGCGGAGTAGCCCGTGTGAGCCCCTTTGGGGGCTTGAAGAGGCTGCCAGCCGGACTTCTGCTGGGTCATGGGCCCATCAGGATGGTCTTGGCGATTCTAGCCTTCTTGAGATTCACGGCAATCAAGCCATCACTGGGTCTCATCAATAGATGGGGTTCAGTGGGGAAAAAAGAGGCTATGGAAATAATAAAGAAGTTCAAGAAAGATCTGGCTGCCATGCTGAGAATAATCAATGCTAGGAAGGAGAAGAAGAGACGAGGCGCAGATACTAATGTCGGAATTGTTGGCCTCCTGCTGACCACAGCTATGGCAGCGGAGGTCACTAGACGTGGGAGTGCATACTATATGTACTTGGACAGAAACGATGCTGGGGAGGCCATATCTTTTCCAACCACATTGGGGATGAATAAGTGTTATATACAGATCATGGATCTTGGACACATGTGTGATGCCACCATGAGCTATGAATGCCCTATGCTGGATGAGGGGGTGGAACCAGATGACGTCGATTGTTGGTGCAACACGACGTCAACTTGGGTTGTGTACGGAACCTGCCATCACAAAAAAGGTGAAGCACGGAGATCTAGAAGAGCTGTGACGCTCCCCTCCCATTCCACTAGGAAGCTGCAAACGCGGTCGCAAACTTGGTTGGAATCAAGAGAATACACAAAGCACTTGATTAGAGTCGAAAATTGGATATTCAGGAACCCTGGCTTCGCGTTAGCAGCAGCTGCCATCGCTTGGCTTTTGGGAAGCTCAACGAGCCAAAAAGTCATATACTTGGTCATGATACTGCTGATTGCCCCGGCATACAGCATCAGGTGCATAGGAGTCAGCAATAGGGACTTTGTGGAAGGTATGTCAGGTGGGACTTGGGTTGATGTTGTCTTGGAACATGGAGGTTGTGTCACCGTAATGGCACAGGACAAACCGACTGTCGACATAGAGCTGGTTACAACAACAGTCAGCAACATGGCGGAGGTAAGATCCTACTGCTATGAGGCATCAATATCGGACATGGCTTCGGACAGCCGCTGCCCAACACAAGGTGAAGCCTACCTTGACAAGCAATCAGACACTCAATATGTCTGCAAAAGAACGTTAGTGGACAGAGGCTGGGGAAATGGATGTGGACTTTTTGGCAAAGGGAGCCTGGTGACATGCGCTAAGTTTGCATGCTCCAAGAAAATGACCGGGAAGAGCATCCAGCCAGAGAATCTGGAGTACCGGATAATGCTGTCAGTTCATGGCTCCCAGCACAGTGGGATGATCGTTAATGACACAGGACATGAAACTGATGAGAATAGAGCGAAGGTTGAGATAACGCCCAATTCACCAAGAGCCGAAGCCACCCTGGGGGGTTTTGGAAGCCTAGGACTTGATTGTGAACCGAGGACAGGCCTTGACTTTTCAGATTTGTATTACTTGACTATGAATAACAAGCACTGGTTGGTTCACAAGGAGTGGTTCCACGACATTCCATTACCTTGGCACGCTGGGGCAGACACCGGAACTCCACACTGGAACAACAAAGAAGCACTGGTAGAGTTCAAGGACGCACATGCCAAAAGGCAAACTGTCGTGGTTCTAGGGAGTCAAGAAGGAGCAGTTCACACGGCCCTTGCTGGAGCTCTGGAGGCTGAGATGGATGGTGCAAAGGGAAGGCTGTCCTCTGGCCACTTGAAATGTCGCCTGAAAATGGATAAACTTAGATTGAAGGGCGTGTCATACTCCTTGTGTACCGCAGCGTTCACATTCACCAAGATCCCGGCTGAAACACTGCACGGGACAGTCACAGTGGAGGTACAGTACGCAGGGACAGATGGACCTTGCAAGGTTCCAGCTCAGATGGCGGTGGACATGCAAACTCTGACCCCAGTTGGGAGGCTGATAACCGCTAACCCCGTAATCACTGAAAGCACTGAGAACTCCAAGATGATGCTGGAACTTGATCCACCATTTGGGGACTCTTACATTGTCATAGGAGTCGGGGAGAAGAAGATCACCCACCACTGGCACAGGAGTGGCAGCACCATTGGAAAAGCATTTGAAGCCACTGTGAGAGGTGCCAGGAGAATGGCAGTCTTGGGAGACACAGCCTGGGACTTTGGATCAGTTGGAGGCGCTCTCAACTCATTGGGCAAGGGCATCCATCAAATTTTTGGAGCAGCTTTCAAATCATTGTTTGGAGGAATGTCCTGGTTCTCACAAATTCTCATTGGAACGTTGCTGATGTGGTTGGGTCTGAACACAAAGAATGGATCTATTTCCCTTATGTGCTTGGCCTTAGGGGGAGTGTTGATCTTCTTATCCACAGCCGTCTCTGCTGATGTGGGGTGCTCGGTGGACTTCTCAAAGAAGGAGACGAGATGCGGTACAGGGGTGTTCGTCTATAACGACGTTGAAGCCTGGAGGGACAGGTACAAGTACCATCCTGACTCCCCCCGTAGATTGGCAGCAGCAGTCAAGCAAGCCTGGGAAGATGGTATCTGTGGGATCTCCTCTGTTTCAAGAATGGAAAACATCATGTGGAGATCAGTAGAAGGGGAGCTCAACGCAATCCTGGAAGAGAATGGAGTTCAACTGACGGTCGTTGTGGGATCTGTAAAAAACCCCATGTGGAGAGGTCCACAGAGATTGCCCGTGCCTGTGAACGAGCTGCCCCACGGCTGGAAGGCTTGGGGGAAATCGTACTTCGTCAGAGCAGCAAAGACAAATAACAGCTTTGTCGTGGATGGTGACACACTGAAGGAATGCCCACTCAAACATAGAGCATGGAACAGCTTTCTTGTGGAGGATCATGGGTTCGGGGTATTTCACACTAGTGTCTGGCTCAAGGTTAGAGAAGATTATTCATTAGAGTGTGATCCAGCCGTTATTGGAACAGCTGTTAAGGGAAAGGAGGCTGTACACAGTGATCTAGGCTACTGGATTGAGAGTGAGAAGAATGACACATGGAGGCTGAAGAGGGCCCATCTGATCGAGATAAAAACATGTGAATGGCCAAAGTCCCACACATTGTGGACAGATGGAATAGAAGAGAGTGATCTGATCATACCCAAGTCTTTAGCTGGGCCACTCAGCCATCACAATACCAGAGAGGGCTACAGGACCCAAATGAAAGGGCCATGGCACAGTGAAGAGCTTGAAATTCGGTTTGAGGAATGCCCAGGCACCAAGGTCCACGTGGAGGAAACATGTGGAACAAGAGGACCATCTCTGAGATCAACCACTGCAAGCGGAAGGGTGATCGAGGAATGGTGCTGCAGGGAGTGCACAATGCCCCCACTGTCGTTCCAGGCTAAAGATGGCTGTTGGTATGGAATGGAGATAAGGCCCAGGAAAGAACCAGAAAGTAACTTAGTAAGGTCAATGGTGACTGCAGGATCAACTGATCACATGGATCACTTCTCCCTTGGAGTGCTTGTGATTCTGCTCATGGTGCAGGAAGGGCTGAAGAAGAGAATGACCACAAAGATCATCATAAGCACATCAATGGCAGTGCTGGTAGCTATGATCCTGGGAGGATTTTCAATGAGTGACCTGGCTAAGCTTGCAATTTTGATGGGTGCCACCTTCGCGGAAATGAACACTGGAGGAGATGTAGCTCATCTGGCGCTGATAGCGGCATTCAAAGTCAGACCAGCGTTGCTGGTATCTTTCATCTTCAGAGCTAATTGGACACCCCGTGAAAGCATGCTGCTGGCCTTGGCCTCGTGTCTTTTGCAAACTGCGATCTCCGCCTTGGAAGGCGACCTGATGGTTCTCATCAATGGTTTTGCTTTGGCCTGGTTGGCAATACGAGCGATGGTTGTTCCACGCACTGATAACATCACCTTGGCAATCCTGGCTGTTCTGACACCACTGGCCCGGGGCACACTGCTTGTGGCGTGGAGAGCAGGCCTTGCTACTTGCGGGGGGTTTATGCTCCTCTCTCTGAAGGGAAAAGGCAGTGTGAAGAAGAACTTACCATTTGTCATGGCCCTGGGACTAACCGCTGTGAGGCTGGTCGACCCCATCAACGTGGTGGGACTGCTGTTGCTCACAAGGAGTGGGAAGCGGAGCTGGCCCCCTAGCGAAGTACTCACAGCTGTTGGCCTGATATGCGCATTGGCTGGAGGGTTCGCCAAGGCAGATATAGAGATGGCTGGGCCCATGGCCGCGGTCGGTCTGCTAATTGTCAGTTACGTGGTCTCAGGAAAGAGTGTGGACATGTACATTGAAAGAGCAGGTGACATCACATGGGAAAAAGATGCGGAAGTCACTGGAAACAGTCCCCGGCTTGATGTGGCGCTAGATGAGAGTGGTGATTTCTCCCTGGTGGAGGATGACGGTCCCCCCATGAGAGAGATCATACTCAAGGTGGTCCTGATGACCATCTGTGGCATGAACCCAATAGCCATACCCTTTGCAGCTGGAGCGTGGTACGTATACGTGAAGACTGGAAAAAGGAGTGGAGCTCTATGGGATGTGCCTGCTCCCAAGGAAGTAAAAAAGGGGGAGACCACAGATGGAGTGTACAGAGTGATGACTCGTAGACTGCTAGGTTCAACACAAGTTGGAGTGGGAGTTATGCAAGAGGGGGTCTTTCACACCATGTGGCACGTCACAAAAGGATCCGCGCTGAGAAGCGGTGAAGGGAGACTTGATCCATACTGGGGAGATGTCAAGCAGGATCTGGTGTCATACTGTGGTCCATGGAAGCTAGATGCCGCCTGGGACGGGCACAGCGAGGTGCAGCTCTTGGCCGTGCCCCCCGGAGAGAGAGCGAGGAACATCCAGACTCTGCCCGGAATATTTAAGACAAAGGATGGGGACATTGGAGCGGTTGCGCTGGATTACCCAGCAGGAACTTCAGGATCTCCAATCCTAGACAAGTGTGGGAGAGTGATAGGACTTTATGGCAATGGGGTCGTGATCAAAAATGGGAGTTATGTTAGTGCCATCACCCAAGGGAGGAGGGAGGAAGAGACTCCTGTTGAGTGCTTCGAGCCTTCGATGCTGAAGAAGAAGCAGCTAACTGTCTTAGACTTGCATCCTGGAGCTGGGAAAACCAGGAGAGTTCTTCCTGAAATAGTCCGTGAAGCCATAAAAACAAGACTCCGTACTGTGATCTTAGCTCCAACCAGGGTTGTCGCTGCCGAAATGGAGGAAGCCCTTAGAGGGCTTCCAGTGCGTTATATGACAACAGCAGTCAATGTCACCCACTCTGGAACAGAAATCGTCGACTTAATGTGCCATGCCACCTTCACTTCACGTCTACTACAGCCAATCAGAGTCCCCAACTATAATCTGTATATTATGGATGAGGCCCACTTCACAGATCCCTCAAGTATAGCAGCAAGAGGATACATTTCAACAAGGGTTGAGATGGGCGAGGCGGCTGCCATCTTCATGACCGCCACGCCACCAGGAACCCGTGACGCATTTCCGGACTCCAACTCACCAATTATGGACACCGAAGTGGAAGTTCCAGAGAGAGCCTGGAGCTCAGGCTTTGATTGGGTGACGGATCATTCTGGAAAAACAGTCTGGTTTGTTCCAAGCGTGAGGAACGGCAATGAGATCGCAGCTTGTCTGACGAAGGCTGGAAAACGGGTCATACAGCTCAGCAGAAAGACTTTTGAGACAGAGTTCCAGAAAACAAAACATCAAGAGTGGGACTTTGTCGTGACAACTGACATTTCAGAGATGGGCGCCAACTTTAAAGCTGACCGTGTCATAGATTCCAGGAGATGCCTAAAGCCGGTCATACTTGATGGCGAGAGAGTCATTCTGGCTGGACCCATGCCTGTCACACATGCCAGCGCTGCCCAGAGGAGGGGGCGCATAGGCAGGAATCCCAACAAACCTGGAGATGAGTATCTGTATGGAGGTGGGTGCGCAGAGACTGACGAAGACCATGCACACTGGCTTGAAGCAAGAATGCTCCTTGACAATATTTACCTCCAAGATGGCCTCATAGCCTCGCTCTATCGACCTGAGGCCGACAAAGTAGCAGCCATTGAGGGAGAGTTCAAGCTTAGGACGGAGCAAAGGAAGACCTTTGTGGAACTCATGAAAAGAGGAGATCTTCCTGTTTGGCTGGCCTATCAGGTTGCATCTGCCGGAATAACCTACACAGATAGAAGATGGTGCTTTGATGGCACGACCAACAACACCATAATGGAAGACAGTGTGCCGGCAGAGGTGTGGACCAGACACGGAGAGAAAAGAGTGCTCAAACCGAGGTGGATGGACGCCAGAGTTTGTTCAGATCACGCGGCCCTGAAGTCATTCAAGGAGTTTGCCGCTGGGAAAAGAGGAGCGGCTTTTGGAGTGATGGAAGCCTTGGGAACACTGCCAGGACACATGACAGAGAGATTCCAGGAAGCCATTGACAACCTCGCTGTGCTCATGCGGGCAGAGACTGGAAGCAGGCCTTACAAAGCCGCGGCGGCCCAATTGCCGGAGACCCTAGAGACCATTATGCTTTTGGGGTTGCTGGGAACAGTCTCGCTGGGAATCTTTTTCGTCTTGATGAGGAACAAGGGCATAGGGAAGATGGGCTTTGGAATGGTGACTCTTGGGGCCAGCGCATGGCTCATGTGGCTCTCGGAAATTGAGCCAGCCAGAATTGCATGTGTCCTCATTGTTGTGTTCCTATTGCTGGTGGTGCTCATACCTGAGCCAGAAAAGCAAAGATCTCCCCAGGACAACCAAATGGCAATCATCATCATGGTAGCAGTAGGTCTTCTGGGCTTGATTACCGCCAATGAACTCGGATGGTTGGAGAGAACAAAGAGTGACCTAAGCCATCTAATGGGAAGGAGAGAGGAGGGGGCAACCATAGGATTCTCAATGGATATTGACCTGCGGCCAGCCTCAGCTTGGGCCATCTACGCTGCCTTGACAACTTTCATTACCCCAGCCGTCCAACATGCAGTGACCACTTCATACAACAACTACTCCTTAATGGCGATGGCCACGCAAGCTGGAGTGTTGTTTGGTATGGGCAAAGGGATGCCATTCTACGCATGGGACTTTGGAGTCCCGCTGCTAATGATAGGTTGCTACTCACAATTAACACCCCTGACCCTAATAGTAGCCATCATTTTGCTCGTGGCGCACTACATGTACTTGATCCCAGGGCTGCAGGCAGCAGCTGCGCGTGCTGCCCAGAAGAGAACGGCAGCTGGCATCATGAAGAACCCTGTTGTGGATGGAATAGTGGTGACTGACATTGACACAATGACAATTGACCCCCAAGTGGAGAAAAAGATGGGACAGGTGCTACTCATAGCAGTAGCCGTCTCCAGCGCCATACTGTCGCGGACCGCCTGGGGGTGGGGGGAGGCTGGGGCCCTGATCACAGCTGCAACTTCCACTTTGTGGGAAGGCTCTCCGAACAAGTACTGGAACTCCTCTACAGCCACTTCACTGTGTAACATTTTTAGGGGAAGTTACTTGGCTGGAGCTTCTCTAATCTACACAGTAACAAGAAACGCTGGCTTGGTCAAGAGACGTGGGGGTGGAACAGGAGAGACCCTGGGAGAGAAATGGAAGGCCCGCTTGAACCAGATGTCGGCCCTGGAGTTCTACTCCTACAAAAAGTCAGGCATCACCGAGGTGTGCAGAGAAGAGGCCCGCCGCGCCCTCAAGGACGGTGTGGCAACGGGAGGCCATGCTGTGTCCCGAGGAAGTGCAAAGCTGAGATGGTTGGTGGAGCGGGGATACCTGCAGCCCTATGGAAAGGTCATTGATCTTGGATGTGGCAGAGGGGGCTGGAGTTACTACGCCGCCACCATCCGCAAAGTTCAAGAAGTGAAAGGATACACAAAAGGAGGCCCTGGTCATGAAGAACCCATGTTGGTGCAAAGCTATGGGTGGAACATAGTCCGTCTTAAGAGTGGGGTGGACGTCTTTCATATGGCGGCTGAGCCGTGTGACACGTTGCTGTGTGACATAGGTGAGTCATCATCTAGTCCTGAAGTGGAAGAAGCACGGACGCTCAGAGTCCTTTCCATGGTGGGGGATTGGCTTGAAAAAAGACCAGGAGCCTTTTGTATAAAAGTGTTGTGCCCATACACCAGCACTATGATGGAAACCCTGGAGCGACTGCAGCGTAGGTATGGGGGAGGACTGGTCAGAGTGCCACTCTCCCGCAACTCTACACATGAGATGTACTGGGTCTCTGGAGCGAAAAGCAACACCATAAAAAGTGTGTCCACCACGAGCCAGCTCCTCTTGGGGCGCATGGACGGGCCCAGGAGGCCAGTGAAATATGAGGAGGATGTGAATCTCGGCTCTGGCACGCGGGCTGTGGTAAGCTGCGCTGAAGCTCCCAACATGAAGATCATTGGTAACCGCATTGAAAGGATCCGCAGTGAGCACGCGGAAACGTGGTTCTTTGACGAGAACCACCCATATAGGACATGGGCTTACCATGGAAGCTATGAGGCCCCCACACAAGGGTCAGCGTCCTCTCTAATAAACGGGGTTGTCAGGCTCCTGTCAAAACCCTGGGACGTGGTGACTGGAGTCACAGGAATAGCCATGACCGACACCACACCGTATGGTCAGCAAAGAGTTTTCAAGGAAAAAGTGGACACTAGGGTGCCAGATCCCCAAGAAGGCACTCGTCAGGTTATGAGCATGGTCTCTTCCTGGTTGTGGAAAGAGCTAGGCAAACACAAACGGCCACGAGTCTGTACCAAAGAAGAGTTCATCAACAAGGTTCGTAGCAATGCAGCATTAGGGGCAATATTTGAAGAGGAAAAAGAGTGGAAGACTGCAGTGGAAGCTGTGAACGATCCAAGGTTCTGGGCTCTAGTGGACAAGGAAAGAGAGCACCACCTGAGAGGAGAGTGCCAGAGTTGTGTGTACAACATGATGGGAAAAAGAGAAAAGAAACAAGGGGAATTTGGAAAGGCCAAGGGCAGCCGCGCCATCTGGTATATGTGGCTAGGGGCTAGATTTCTAGAGTTCGAAGCCCTTGGATTCTTGAACGAGGATCACTGGATGGGGAGAGAGAACTCAGGAGGTGGTGTTGAAGGGCTGGGATTACAAAGACTCGGATATGTCCTAGAAGAGATGAGTCGCATACCAGGAGGAAGGATGTATGCAGATGACACTGCTGGCTGGGACACCCGCATCAGCAGGTTTGATCTGGAGAATGAAGCTCTAATCACCAACCAAATGGAGAAAGGGCACAGGGCCTTGGCATTGGCCATAATCAAGTACACATACCAAAACAAAGTGGTAAAGGTCCTTAGACCAGCTGAAAAAGGGAAGACAGTTATGGACATTATTTCGAGACAAGACCAAAGGGGGAGCGGACAAGTTGTCACTTACGCTCTTAACACATTTACCAACCTAGTGGTGCAACTCATTCGGAGTATGGAGGCTGAGGAAGTTCTAGAGATGCAAGACTTGTGGCTGCTGCGGAGGTCAGAGAAAGTGACCAACTGGCTGCAGAGCAACGGATGGGATAGGCTCAAACGAATGGCAGTCAGTGGAGATGATTGCGTTGTGAGGCCAATTGATGATAGGTTTGCACATGCCCTCAGGTTCTTGAATGATATGGGGAAAGTTAGGAAGGACACACAAGAGTGGAAACCCTCAACTGGATGGGACAACTGGGAGGAAGTTCCGTTTTGCTCCCACCACTTCAACAAGCTCCATCTCAAGGACGGGAGGTCCATTGTGGTTCCCTGCCGCCACCAAGATGAACTGATTGGCCGGGCCCGCGTCTCTCCAGGGGCGGGATGGAGCATCCGGGAGACTGCTTGCCTAGCAAAATCATATGCGCAAATGTGGCAGCTCCTTTATTTCCACAGAAGGGACCTCCGACTGATGGCCAATGCCATTTGTTCATCTGTGCCAGTTGACTGGGTTCCAACTGGGAGAACTACCTGGTCAATCCATGGAAAGGGAGAATGGATGACCACTGAAGACATGCTTGTGGTGTGGAACAGAGTGTGGATTGAGGAGAACGACCACATGGAAGACAAGACCCCAGTTACGAAATGGACAGACATTCCCTATTTGGGAAAAAGGGAAGACTTGTGGTGTGGATCTCTCATAGGGCACAGACCGCGCACCACCTGGGCTGAGAACATTAAAAACACAGTCAACATGGTGCGCAGGATCATAGGTGATGAAGAAAAGTACATGGACTACCTATCCACCCAAGTTCGCTACTTGGGTGAAGAAGGGTCTACACCTGGAGTGCTGTAAGCACCAATCTTAGTGTTGTCAGGCCTGCTAGTCAGCCACAGCTTGGGGAAAGCTGTGCAGCCTGTGACCCCCCCAGGAGAAGCTGGGAAACCAAGCCTATAGTCAGGCCGAGAACGCCATGGCACGGAAGAAGCCATGCTGCCTGTGAGCCCCTCAGAGGACACTGAGTCAAAAAACCCCACGCGCTTGGAGGCGCAGGATGGGAAAAGAAGGTGGCGACCTTCCCCACCCTTTAATCTGGGGCCTGAACTGGAGATCAGCTGTGGATCTCCAGAAGAGGGACTAGTGGTTAGAGGAGACCCCCCGGAAAACGCAAAACAGCATATTGACGCTGGGAAAGACCAGAGACTCCATGAGTTTCCACCACGCTGGCCGCCAGGCACAGATCGCCGAATAGCGGCGGCCGGTGTGGGGAAATCCATGGGTCTGGGTCGGCATGGCATCTCCACCTCCTCGCGGTCCGACCTGGGCTACTTCGGTAGGCTAAGGGAGAAGCTCGAGCCAGCTTTTGTTCCCTTTAGTGAGGGTTAATTGCGCGCTTGGCGTAATCATGGTCATAGCTGTTTCCTGTGTGAAATTGTTATCCGCTCACAATTCCACACAACATACGAGCCGGAAGCATAAAGTGTAAAGCCTGGGGTGCCTAATGAGTGAGCTAACTCACATTAATTGCGTTGCGCTCACTGCCCGCTTTCCAGTCGGGAAACCTGTCGTGCCAGCTGCATTAATGAATCGGCCAACGCGCGGGGAGAGGCGGTTTGCGTATTGGGCGCTCTTCCGCTTCCTCGCTCACTGACTCGCTGCGCTCGGTCGTTCGGCTGCGGCGAGCGGTATCAGCTCACTCAAAGGCGGTAATACGGTTATCCACAGAATCAGGGGATAACGCAGGAAAGAACATGTGAGATCTCTACGGGTCGGATTTGAAGTCGTCTTGGTAGGAGGCAGCCTGAATGGCGAATGCCGATGCCCTTGAGAGCCTTCAACCCAGTCAGCTCCTTCCGGTGGGCGCGGGGCATGACTATCGTCGCCGCACTTATGACTGTCTTCTTTATCATGCAACTCGTAGGACAGGGTGCCGGCAGCGCTCTGGGTCATTTTCGGCGAGGACCGCTTTCGCTGGAGCGCGACGATGATCGGCCTGTCGCTTGCGGTATTCGGAATCTTGCACGCCCTCGCTCAAGCCTTCGTCACTGGTCCCGCCACCAAACGTTTCGGCGAGAAGCAGGCCATTATCGCCGGCATGGCGGCCGACGCGCTGGGCTACGTCTTGCTGGCGTTCGCGACGCGAGGCTGGATGGCCTTCCCCATTATGATTCTTCTCGCTTCCGGCGGCATCGGGATGCCCGCGTTGCAGGCCATGCTGTCCAGGCAGGTAGATGACGACCATCAGGGACAGCTTCAAGGATCGCTCGCGGCTCTTACCAGCCTAACTTCGATCATTGGACCGCTGATCGTCACGGCGATTTATGCCGCCTCGGCGAGCACATGGAACGGGTTGGCATGGATTGTAGGCGCCGCCCTATACCTTGTCTGCCTCCCCGCGTTGCGTCGCGGTGCATGGAGCCGGGCCACCTCGACCTGAATGGAAGCCGGCGGCACCTCGCTAACGGATTCACCACTCCGCAGACCCGCCATAAAACGCCCTGAGAAGCCCGTGACGGGCTTTTCTTGTATTATGGGTAGTTTCCTTGCATGAATCCATAAAAGGCGCCTGTAGTGCCATTTACCCCCATTCACTGCCAGAGCCGTGAGCGCAGCGAACTGAATGTCACGAAAAAGACAGCGACTCAGGTGCCTGATGGTCGGAGACAAAAGGAATATTCAGCGATTTGCCCGAGCTTGCGAGGGTGCTACTTAAGCCTTTAGGGTTTTAAGGTCTGTTTTGTAGAGGAGCAAACAGCGTTTGCGACATCCTTTTGTAATACTGCGGAACTGACTAAAGTAGTGAGTTATACACAGGGCTGGGATCTATTCTTTTTATCTTTTTTTATTCTTTCTTTATTCTATAAATTATAACCACTTGAATATAAACAAAAAAAACACACAAAGGTCTAGCGGAATTTACAGAGGGTCTAGCAGAATTTACAAGTTTTCCAGCAAAGGTCTAGCAGAATTTACAGATACCCACAACTCAAAGGAAAAGGACTAGTAATTATCATTGACTAGCCCATCTCAATTGGTATAGTGATTAAAATCACCTAGACCAATTGAGATGTATGTCTGAATTAGTTGTTTTCAAAGCAAATGAACTAGCGATTAGTCGCTATGACTTAACGGAGCATGAAACCAAGCTAATTTTATGCTGTGTGGCACTACTCAACCCCACGATTGAAAACCCTACAAGGAAAGAACGGACGGTATCGTTCACTTATAACCAATACGCTCAGATGATGAACATCAGTAGGGAAAATGCTTATGGTGTATTAGCTAAAGCAACCAGAGAGCTGATGACGAGAACTGTGGAAATCAGGAATCCTTTGGTTAAAGGCTTTGAGATTTTCCAGTGGACAAACTATGCCAAGTTCTCAAGCGAAAAATTAGAATTAGTTTTTAGTGAAGAGATATTGCCTTATCTTTTCCAGTTAAAAAAATTCATAAAATATAATCTGGAACATGTTAAGTCTTTTGAAAACAAATACTCTATGAGGATTTATGAGTGGTTATTAAAAGAACTAACACAAAAGAAAACTCACAAGGCAAATATAGAGATTAGCCTTGATGAATTTAAGTTCATGTTAATGCTTGAAAATAACTACCATGAGTTTAAAAGGCTTAACCAATGGGTTTTGAAACCAATAAGTAAAGATTTAAACACTTACAGCAATATGAAATTGGTGGTTGATAAGCGAGGCCGCCCGACTGATACGTTGATTTTCCAAGTTGAACTAGATAGACAAATGGATCTCGTAACCGAACTTGAGAACAACCAGATAAAAATGAATGGTGACAAAATACCAACAACCATTACATCAGATTCCTACCTACATAACGGACTAAGAAAAACACTACACGATGCTTTAACTGCAAAAATTCAGCTCACCAGTTTTGAGGCAAAATTTTTGAGTGACATGCAAAGTAAGTATGATCTCAATGGTTCGTTCTCATGGCTCACGCAAAAACAACGAACCACACTAGAGAACATACTGGCTAAATACGGAAGGATCTGAGGTTCTTATGGCTCTTGTATCTATCAGTGAAGCATCAAGACTAACAAACAAAAGTAGAACAACTGTTCACCGTTACATATCAAAGGGAAAACTGTCCATATGCACAGATGAAAACGGTGTAAAAAAGATAGATACATCAGAGCTTTTACGAGTTTTTGGTGCATTCAAAGCTGTTCACCATGAACAGATCGACAATGTAACAGATGAACAGCATGTAACACCTAATAGAACAGGTGAAACCAGTAAAACAAAGCAACTAGAACATGAAATTGAACACCTGAGACAACTTGTTACAGCTCAACAGTCACACATAGACAGCCTGAAACAGGCGATGCTGCTTATCGAATCAAAGCTGCCGACAACACGGGAGCCAGTGACGCCTCCCGTGGGGAAAAAATCATGGCAATTCTGGAAGAAATAGCGCTTTCAGCCGGCAAACCGGCTGAAGCCGGATCTGCGATTCTGATAACAAACTAGCAACACCAGAACAGCCCGTTTGCGGGCAGCAAAACCCGTACTTTTGGACGTTCCGGCGGTTTTTTGTGGCGAGTGGTGTTCGGGCGGTGCGCGCAAGATCCATTATGTTAAACGGGCGAGTTTACATCTCAAAACCGCCCGCTTAACACCATCAGAAATCCTCAGCGCGATTTTAAGCACCAACCCCCCCCCGTAACACCCAAATCCATACTGAAAGTGGCTTTGTTGAATAAATCAGATTTCGGGTAAGTCTCCCCCGTAGCGGGTTGTGTTTTCAGGCAATACGCACGCTTTCAGGCATACCTGCTTTCGTCATTTTGTTCAGCGCTCGTACCAGGGCCATAGCCTCCGCAACCTGACCATCGTAGTCACGCAGCGTCAGTGAACCCCCGAACAGAGA
